# Evaluating patient safety research performance in Arab world countries: Changing trends and reflections

**DOI:** 10.12669/pjms.39.6.7514

**Published:** 2023

**Authors:** Nadeem Alam Zubairi, Nadeem Shafique Butt, Ahmad Azam Malik, Zohair Jamil Gazzaz

**Affiliations:** 1 Nadeem Alam Zubairi, Department of Pediatrics, Faculty of Medicine in Rabigh, King Abdulaziz University, Jeddah, Saudi Arabia; 2 Nadeem Shafique Butt, Department of Family and Community Medicine, Faculty of Medicine in Rabigh, King Abdulaziz University, Jeddah, Saudi Arabia; 3 Ahmad Azam Malik, Department of Family and Community Medicine, Faculty of Medicine in Rabigh, King Abdulaziz University, Jeddah, Saudi Arabia; 4 Zohair Jamil Gazzaz, Department of Medicine, Faculty of Medicine in Rabigh, King Abdulaziz University, Jeddah, Saudi Arabia

**Keywords:** Patient Safety, Medical Research, Bibliometric analyses, Arab World

## Abstract

**Background and Objective:**

Patient safety is a major concern in health care. Research is an important tool to minimize preventable errors. Research performance and trends evaluation need to be identified for future guidance. Our objective was to evaluate the research performance in Arab World countries related to patient safety so that real picture is available to all stake holders for future application

**Methods:**

This was a descriptive exploratory study carried at King Abdulaziz University Jeddah, using Bibliometric analyses on Web of Science extracted data, exploring the research publications related to Patient Safety from the Arab World in last two decades (2001-2020). Digital resources were used. Data collected was further explored to see the trends.

**Results:**

Only 2% of total worldwide publications on Patient Safety were from Arab World. A positive trend, however, has emerged since 2015. Out of 5940 documents identified, only 383 had single authorship. Egypt and Saudi Arab were the major contributors. Other countries had less or even zero publications. Researchers are coordinating with others in Western countries to enhance the research productivity. Cairo University with 734 publications had most affiliations. Publications on safety culture and medication safety were frequent. Hospital Acquired Infections and error reporting had limited research.

**Conclusion:**

Researches on patient safety in the Arab World are not sufficient. Countries other than Egypt and Saudi Arabia also need to contribute more frequently. Critical problems, like Hospital Acquired Infections, should have regular research from all countries to assist those treating patients and those making health related policies.

## INTRODUCTION

According to WHO, patient safety (PS) means absence of preventable harm to a patient throughout health care delivery process and minimizing its associated risk of unnecessary harm.^1^ Any negligence from health delivery team is termed as medical error and it directly compromises the safety of the patients under care. Patient safety emphasizes safety in health care brought not only through prevention and reduction of errors but also by better reporting and analysis of these.

Although the concept of safety while taking care of patients and doing no harm is known since the days of Hippocrates but it was not until 1990s that the magnitude of the problem was genuinely recognized following several reports in the USA where medical related errors are the 3^rd^ leading cause of mortality after heart disease and cancer.^2^ Since then, all around the world there is more awareness and realization to implement safety culture in all medical facilities.^3^ The situation in the Arab World countries is no different.^4^

To counter any existing or growing problem, the best strategy is to do research. The scientific literature on different health issues, including PS, is usually overwhelming, increasing the demand from all stakeholders including the researchers to access the real picture.^5^

A lot of research has been generated in the USA, England and China related to patient safety and substantial work has been done in other countries.^6^ Unfortunately, in Arab World the biomedical research is presumed to be relatively less than few other parts of the world.^7^ Subsequently, literature too is scarcely available on research performance and trends evaluation from the Arab region. The aim of our study was to analyze all publications from the Arab World in last two decades (2001-2020) related to Patient Safety. It is critical to evaluate the research performance in this area and identify trends, strengths, and limitations in order to plan and assist in future investments besides guiding future researchers. It is needed to help the policy makers to formulate plans for minimizing these essentially preventable deaths and morbidities.

## METHODS

This was a descriptive exploratory study carried out at King Abdulaziz University Jeddah, Kingdom of Saudi Arabia (KSA). It was designed to provide a concise but complete overview of Patient Safety associated publications done in the Arab World during the last two decades starting from year 2001. Web of Science (WoS), being the most commonly used relatively credible and relevant database, was chosen to retrieve the relevant documents. The WoS is a Clarivate Analytics (Thomson Reuters, formerly) maintained platform and is considered as a comprehensive source for exploration of scientific documents having the highest quality indexing.^8^ King Abdul Aziz University (KAU) digital resources and online library were used to collect the data from Web of Science Database, access to which is permitted. This research employed scientometric techniques. Efforts were made to assure the quality of data not only at the initial extraction step but also at later processing phases. It analyzed all the published documents focusing on Patient Safety between 2001 and 2020 in WoS. The following search strategy was used: TS=Patient safety AND CU= (ALGERIA OR BAHRAIN OR CAMEROON OR DJIBOUTI OR EGYPT OR IRAQ OR JORDAN OR KUWAIT OR LEBANON OR LIBYA OR MAURITANIA OR MOROCCO OR OMAN OR PALESTINE OR QATAR OR SAUDI ARABIA OR SOMALIA OR SUDAN OR SYRIA OR TUNISIA OR UNITED ARAB EMIRATES OR YEMEN).

### TOPIC

(“Patient Safety”) Refined by Countries (CU): (names of all Arab World Countries*) Timespan: 2001–2020. Indexes: SCIEXPANDED, SSCI, A&HCI, CPCI-S, CPCI-SSH, ESCI.

*Arab World means all the 22 countries defined by the Arab League.^9^ No document type and language limitations were applied. The search was conducted on 18^th^ December 2021. All types of publications were initially included and were later scrutinized manually by two researchers independently to exclude irrelevant documents.

Data was extracted from WoS in plain text files and after excluding irrelevant documents beyond the study scope, bibliometric analysis at the document, source and author levels was performed by using the “R-Bibliometrix” package.^10^ Our search resulted in identifying 6103 publications. A total of 163 papers were found irrelevant and therefore invalid for inclusion. Thus, we had a total of 5940 documents relevant to our topic that were selected for further detailed analysis. We employed a wide range of bibliometrics indicators including publication output and growth trend, authors and their institutional affiliations, journals publishing on patient safety, countries distribution, citation analysis and collaboration networks. Two researchers (AAM and NSB) independently searched and extracted the required information to verify the process while the other two researchers (NAZ and ZJG) separately did the manual check to exclude irrelevant documents. Considering the diversity of issues related to PS, a further analysis of extracted documents was done to see the topic-wise preferences and trends.

## RESULTS

**Fig.1:**
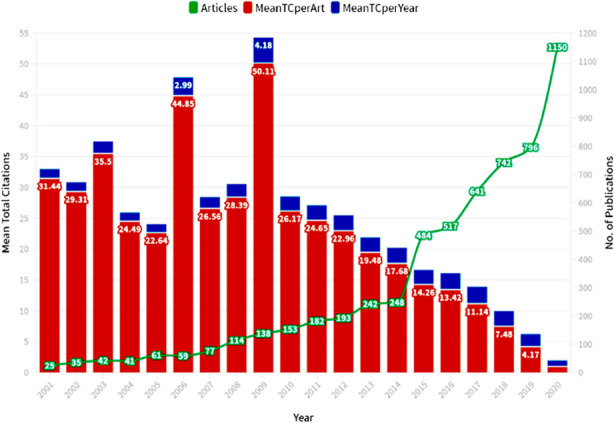
Publications and mean total citations.

A total of 5940 documents were relevant to our topic and selected for further detailed analysis. A summary of these articles is given in Table-I. The trend in publications and citations over these last two decades are shown in Fig.1. Most of the documents related to PS from Arab Region have multiple authors. Only 6.4% documents had single authorship. Countries with most contributions are summarized in Table-II.

**Table-I:** Summary of Research on Patient Safety in Arab World

Description	2001-2020
Documents	5940
Annual growth rate (%)	22.23%
Open access	2510
Sources (Journals, Books, etc.)	1858
Average years from publication	5.16
Average citations per documents	12.38
Average citations per year per doc	1.726
References	160764
***Document Contents***	
Keywords Plus (ID)	12067
Author’s Keywords (DE)	12538
Authors	35355
Author Appearances	49670
***Authors Collaboration***	
Single-authored documents	383
Documents per Author	0.168
Authors per Document	5.95
Co-Authors per Documents	8.36
Collaboration Index	6.3
***Document Types***	
Articles	4726
Editorials	46
Letters/Correspondences	28
Reviews	845
Others	295

**Table-II:** Top 10 countries productivity

Country	CA	Author Appearances	Percentage Contribution	SCP	MCP	MCP Ratio	TC	Average Article Citations
EGYPT	1625	4586	28.2%	1383	242	0.149	12947	7.97
SAUDI ARABIA	985	3292	17.1%	653	332	0.337	7092	7.2
USA	467	3392	8.1%	5	462	0.989	15396	32.97
LEBANON	283	1069	4.9%	157	126	0.445	4404	15.56
UK	187	1344	3.2%	4	183	0.979	3411	18.24
UAE	186	502	3.2%	87	99	0.532	1217	6.54
CANADA	179	1043	3.1%	1	178	0.994	3496	19.53
JORDAN	174	519	3.0%	128	46	0.264	1293	7.43
QATAR	166	564	2.9%	76	90	0.542	1138	6.86
FRANCE	145	958	2.5%	24	121	0.834	2486	17.14

CA - Corresponding author, SCP: Single or Intra-country publication, MCP: Multiple or Inter-country publications, TC – Total citations.

**Fig.2:**
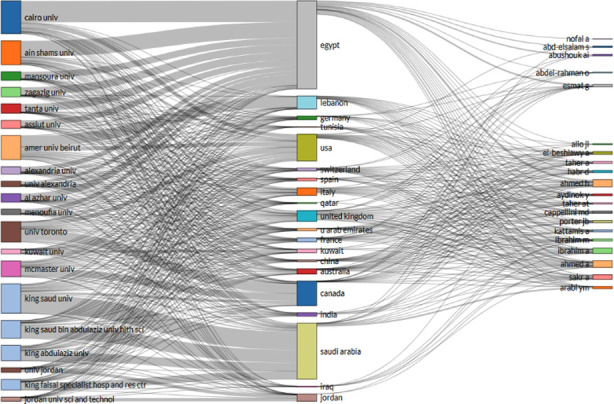
Three Field Plot for top 20 most productive affiliations, countries and authors.

Altogether, 7671 institutes contributed to produce 5940 publications in the study scope. The collaborative index was 6.3. Six of the top 10 contributory universities were from Egypt, headed by Cairo University (734 Publications) while three were from KSA, headed by King Saud University (551 publications). American University of Beirut was the other institution among top ten, having 435 publications from it. Fig.2 shows a three-field plot for the top 20 most productive affiliated organizations, countries and authors.

**Fig.3:**
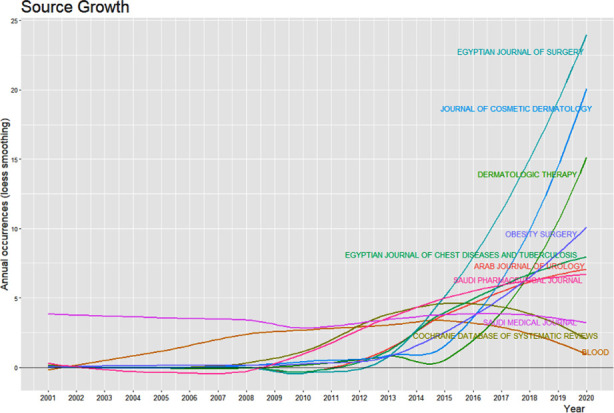
Year-wise growth of 10 most productive sources.

A total of 1858 sources were identified for the documents obtained. The top ten sources and their growth in last two decades is shown in Fig.3. Egyptian Journal of Surgery, with 101 publications, heads the list followed by Saudi Medical Journal (77 publications). While few of the journals have grown in publications related to PS sharply in last five years, Saudi Medical Journal is maintaining a constant number of PS related articles since 2001. Extracted documents were further analyzed according to the topic or PS issue under discussion. These had two categories:

- Documents on general themes and issues related to Patient Safety (805 documents, 13.5%)
- Documents related to use and safety of specific medicines or surgical procedures including comparative studies between medicines and procedures (5135 documents, 86.5%)

The first group was further scrutinized to explore the topics covered and the results were as shown in Table-III.

**Table-III:** Number of documents on different Patient Safety issues.

Topics*	Documents
Safety Culture and factors influencing PS	288
Medication safety and Adverse Drug Reactions	99
Patient Safety issues related to Nursing	90
Information Technology (IT) and e-Health related to Patient Safety	68
Community and Hospital Pharmacists	65
Hospital Acquired Infections	56
Reporting of Adverse Events	39
Safety issues in Emergencies and Emergency Rooms	29
COVID-19 and Patient Safety	28
Patient Safety issues in Intensive Care Units	25
General Surgery and Patient Safety	20
Blood Transfusion related Patient Safety issues	19
Patient Safety concerns in Outpatient Department	04
Patient Safety issues related to Routine Vaccination	03

* There are few overlaps. For example, “DOES CPOE SUPPORT NURSE-PHYSICIAN COMMUNICATION IN THE MEDICATION ORDER PROCESS? A NURSING PERSPECTIVE”, covers PS issues related to both the use of IT in medicine and Nursing.

## DISCUSSION

Our study revealed that for research activity on patient safety, the contribution of Arab World was a meager 2% of the global activity (Only 5940 out of a total of 302,869 documents related to PS published throughout the world from 2001 to 2020). None of the countries from Arab World are in the top 30 countries in this regard. The USA, China, UK, Germany and Japan are major contributors.^11^ However, a positive trend has been noticed. Though still not matching the global trend but there has been a sharp increase in research publications related to and involving PS issues from the Arab World Countries since start of 2015. The period between 2015 to 2020 has a share of 73% of the total PS related publications in the two decades starting from 2001, as evident in Fig.1. This trend of including PS issues in research is indicative of awareness among doctors, nurses and pharmacists of the Arab region about the magnitude and importance of the subject

Combined research and sharing of results are helping researchers to generate bigger data covering larger populations while increasing their research authenticity, output and profile.^12^ Most of the publications in our study had multiple authors (Average 5.95 per publication). Only 383 publications (6.4%) had single authorship. On further scrutiny of top 10 authors, it was revealed that six out of 10 top productive authors are hematologists working in their respective countries but are doing collaborative research activities together. Hence, in most of their publications they were co-authors.

Comparing the statistics of Dany Habr (28 publications) with Omar Abdel Rahman (27 publications), it was noted that while Omar was first author in 19 and corresponding author in 21 of his publications, the former researcher was neither the first author nor the corresponding author in any of his 28 publications. This highlights the variability in contributions among co-authors in a research paper.

Research productivity on PS is very variable among different Arab countries. Egypt and Kingdom of Saudi Arabia were the major contributors. It can be noted that researchers from countries outside the Arab Region also contributed significantly due to mutual collaboration. The other five countries with relatively better productivity were Lebanon, UAE, Jordan, Qatar and Kuwait. Remaining 13 Arab countries, not in the Table-III but mentioned in methodology, had much lesser studies published. While we didn’t find any paper from Djibouti, there were less than 10 papers from Somalia, Cameroon and Mauritania in the last two decades on PS. Syria, Libya, Yemen and Palestine all had less than 50 publications related to our scope, which might be due to political instability in these countries during the last two decades. We recommend all Arab countries to have more frequent researches on all aspects of Patient Safety. This will assist them in knowing the local status and will help heath providers and policy makers to adopt recommended changes

Egypt, Saudi Arabia and Jordan are all showing collaboration with all the top 20 universities irrespective of these belonging to their respective country or not, as evident from Fig.2. This affiliation is, however, not present with prolific authors mentioned earlier. Only Egypt has association/collaboration with these most contributing authors, irrespective of their country of origin.

Citations have been presumed to reflect the impact of any research or its quality. Over the last two decades, the importance of citations as performance indicators in research system has increased tremendously and, in a way, dented other dimensions of research quality like originality, solidity and societal significance.^13^,^14^ Researchers are being judged and rewarded accordingly while journals are dependent on citations for their Impact Factor. In our study, the most cited research paper was, “A Surgical Safety Checklist to Reduce Morbidity and Mortality in a Global Population”. In this global research, Arab world was represented by Jordan. However, on the basis of Internal Citations, the most cited study was, “The current state of patient safety culture in Lebanese hospitals: a study at baseline” from American University of Beirut, Beirut, Lebanon.

We identify that 13.5% of identified publications from the Arab World were related to general themes and issues concerning Patient Safety while rest were related to the use and safety of specific medicines or surgical procedures including comparative studies between medicines and procedures. In the first group, most publications were related to presence and implementation of safety culture in health care institutions and factors influencing PS, as shown in Table-III. Among these, there were 25 documents related to PS issues in Dentistry, eight on effects of Accreditation of health institutes on PS, three on PS being compromised specifically due to ‘language barriers’ while two papers were published on use of ‘abbreviations’ effecting safety of patients.

Nurses are an important stakeholder in PS concerns. Their adherence to PS principles is one of the important keys for avoiding medical errors.^15^ We found 90 publications from the Arab World related to PS and Nurses, mostly dealing with effects on PS due to workload/work environment while issues like substance abuse were also investigated.

Use of Information Technology (IT) in medicine has gradually increased in the last two decades and has become part and parcel of the health delivery system of today. Despite huge advantages, researchers have advised a balance approach to ensure patient safety related to use of IT.^16^ In Arab World too, the researchers have explored the various modalities of IT being used in patient care and their safety.^17^ There were 68 publications on the subject during the study period, examining their benefits and potential risks. These covered a variety of related topics including electronic prescription and records, computer literacy among health givers, robotic nursing, cloud and smart fog computing in patient care, etc. Researchers have also investigated the use and safety of Radio-frequency identification (*RFID*), Computerized provider order entry (CPOE), Bar Coded Medication Administration (*BCMA*) and Medication exchange and sharing network program (MESNP). There were only two papers on safety of Artificial Intelligence (AI) in medicine.^18^

Community and hospital pharmacist have to play an important role in ensuring PS. Over the decades, the pharmacists are contributing in publications related to their field including PS aspects.^19^ We extracted 65 publications from Arab region, during the study period, pertaining to Pharmacists’ role in PS. Fifty-five of these (85%) were after 2015, indicating a recent trend. Interestingly, these publications were from many countries including those like Sudan and Algeria, who had otherwise relatively little contribution in PS related publications. Most publications were from KSA and Jordan, covering a lot of topics from safe pharmacy practices to topics like role of pharmacists in helping visually impaired persons.^20^,^21^

Hospital Acquired Infections remains a great threat. Seven admitted patients out of 100 in high income countries and 10 out of 100 in low-income countries are getting HAI, including highly resistant infections.^22^ During the last two decades, 56 papers were published on HAI related to Arab World. Considering the gravity of the issue and contemporary research on the subject in the world, these are less than expected. Is this trend going to deprive us from getting current status and new trends? We suggest publishing of meta-analysis of data related to important issues like HAI to be mandatory for every country.

*Reporting* errors is a fundamental requirement to prevent errors.^23^ In Arab World too, the numbers of reported cases of medical neglect are on the rise.^24^ As the implementation of safety culture is improving so is the trend to report an error in health delivery system by the members of that system.^25^ Our search identified 39 papers raising the issue of reporting medical errors in the Arab World in the last two decades. Most of these were from Saudi Arabia. We recommend that other countries of the region should explore their status through research so that a culture of reporting errors can be implemented effectively.

### Limitations

Our study has the limitation of being based only on WoS extracted data. Therefore, some limitation in the generalizability of the findings can be considered. Secondly, continuous updates and resultant changes in WoS data may result in slightly different publication data depending upon the date of search and time frame. Analyses from other sources with different time frames might be complementary to this study and may provide a more comprehensive picture on the subject.

## CONCLUSION

Patient Safety is a major health care concern. Related publications from Arab World Countries in the last two decades however have been meagre, contributing only 2% of the total. A positive trend has emerged since 2015, though it still is far from expected when we compare it with similar global activity. Egypt and Saudi Arabia are leading contributors in Arab World while many countries are contributing minimally. There is a trend among Arab researchers to coordinate with each other and with researchers in other parts of the world. Single authorship publications were only 6.5%. Few issues related to patient safety like implementation of safety culture, medication safety and role of nurses have relatively good number of publications. Contribution from pharmacists too was substantial. Studies related to Hospital Acquired Infections are few or none from most of the countries

### Recommendations

To comprehend their own local status and to minimizing adverse events, researchers in Arab countries other than Egypt and Saudi Arabia, also need to contribute more frequently on issues related to PS. Trend of reporting a medical error remains unknown in most of the Arab World due to lack of related research and should be addressed. We also recommend that critical issues, like Hospital Acquired Infections, should have regular research from all countries to help out those treating patients and those making health related policies.

### Authors’ Contributions:

**NAZ:** Conceptualization, Manual check of data, preparing the draft, work integrity and accuracy. He is also responsible for the accuracy of the study.

**NSB:** Data extraction and analysis of data, Manuscript writing, work integrity and accuracy.

**AAM:** Data extraction and analysis of data, Manuscript writing, work integrity and accuracy.

**ZJG:** Manual check of data, Critical revision of Manuscript, work integrity and accuracy.
